# Retrospective analysis of the success and safety of Gold Micro Shunt Implantation in glaucoma

**DOI:** 10.1186/1471-2415-13-35

**Published:** 2013-07-18

**Authors:** Arno Hueber, Sigrid Roters, Jens F Jordan, Walter Konen

**Affiliations:** 1 Department of Ophthalmology, University of Cologne, Kerpener Str. 62, 50937 Cologne, Germany; 2 University Eye Hospital Freiburg, 79106 Freiburg, Germany

**Keywords:** Glaucoma, Glaucoma drainage implants, Gold micro shunt, Shunt implantation to the supraciliary space

## Abstract

**Background:**

To evaluate the success rate and adverse effects of Gold Micro Shunt Plus (GMS+) implantation into the supraciliary space.

**Methods:**

This retrospective study included 31 eyes of 31 patients diagnosed with severe glaucoma and uncontrolled intraocular pressure (IOP) with implantation of a GMS+ by means of a full-thickness scleral flap. The main outcome measures were surgical failure or success, based on the intraocular pressure and adverse effects. Clinical examination data are reported up to 4 years postoperatively.

**Results:**

Thirty eyes (97%) met one of our criteria for failure. Within a mean of 7.3 ± 7.7 months another surgery was performed because of elevated IOP in 24 of 31 eyes (77%) and because of adverse effects in 2 (6%). The remaining 4 eyes, that met one of our criteria for failure, had an IOP reduction of less than 20% with comparable medication. Six GMS+’s were explanted; because of IOP elevation, 2; rubeosis iridis, 2; and low grade inflammation, 2.

**Conclusions:**

GMS+ implantation is not an effective method to control IOP in patients with glaucoma, when using our surgical technique. The reason for the found signs of chronic low grade inflammation or rubeosis iridis in 4 eyes (13%) remains unknown and has to be further investigated.

## Background

A recent, alternative approach to treating glaucoma with filtration procedures, such as trabeculectomy, is the drainage of aqueous humor from the anterior chamber (AC) to the suprachoroidal space. This approach avoids the conjunctiva, which is known to be responsible for the failure of trabeculectomies. Another advantage to this shunt route is that the pressure in the suprachoroidal space serves as a natural counter pressure to prevent severe postoperative hypotony. Emi et al. [1] found a drop in hydrostatic pressure from the anterior chamber to the suprachoroidal space up to -3.7 ± 0.4 mmHg that increased with experimentally raised intraocular pressure. This negative pressure in the suprachoroidal space provides the rational for a shunt from the AC to the suprachoroidal space to lower the intraocular pressure (IOP). Aqueous filtration across the sclera may be another possible outflow pathway [2].

We reported our encouraging experience with a silicone shunt which connected the anterior chamber to the suprachoroidal space [3]. We then exploited the advantages of this novel shunt route, but replaced silicone with gold as the shunt material, seeking a more stable and safe IOP reduction. Therefore we evaluated this recent device for suprachoroidal drainage, the Gold Micro Shunt Plus (GMS+).

## Methods

This was a retrospective study of patients with uncontrolled intraocular pressure who underwent GMS+ implantation from February 2006 to June 2008 at the Department of Ophthalmology, University of Cologne, Germany. Our local Ethic Board was notified and determined that the board’s approval was not necessary in a retrospective study.

### Shunt design

The GMS+ (SOLX ltd, Waltham, Massachusetts) is a recently developed device for suprachoroidal drainage. According to the manufacturer, the implant is made of biocompatible, 99.95% 24-carat gold. The GMS+ is a thin plate which measures 5.2 mm in length, 3.2 mm in width and 68 μm in height. Nine or nineteen micro channels in the GMS+ have a height of 40 μm and a width of 50 μm. We could not determine which of the GMS+ shunts used 9 or 19 microchannels because the design of the GMS+ changed and the manufacture did not provide us lot-specific design information.

#### *Data collection*

Preoperative data collected from the patient records included age at the time of surgery, IOP on the day prior to GMS+ implantation, prior ocular surgical procedures, specific glaucoma diagnosis and other ocular history, IOP lowering medications, and Cup-disc ratio.

Postoperative data were collected from the patient records from all consecutive visits. Collected data included IOP measurements, IOP lowering medications, surgical complications, additional surgical procedures performed, and follow-up time. Clinical examination data were reported up to 4 years postoperatively.

#### *Surgical technique*

**Figure 1:**
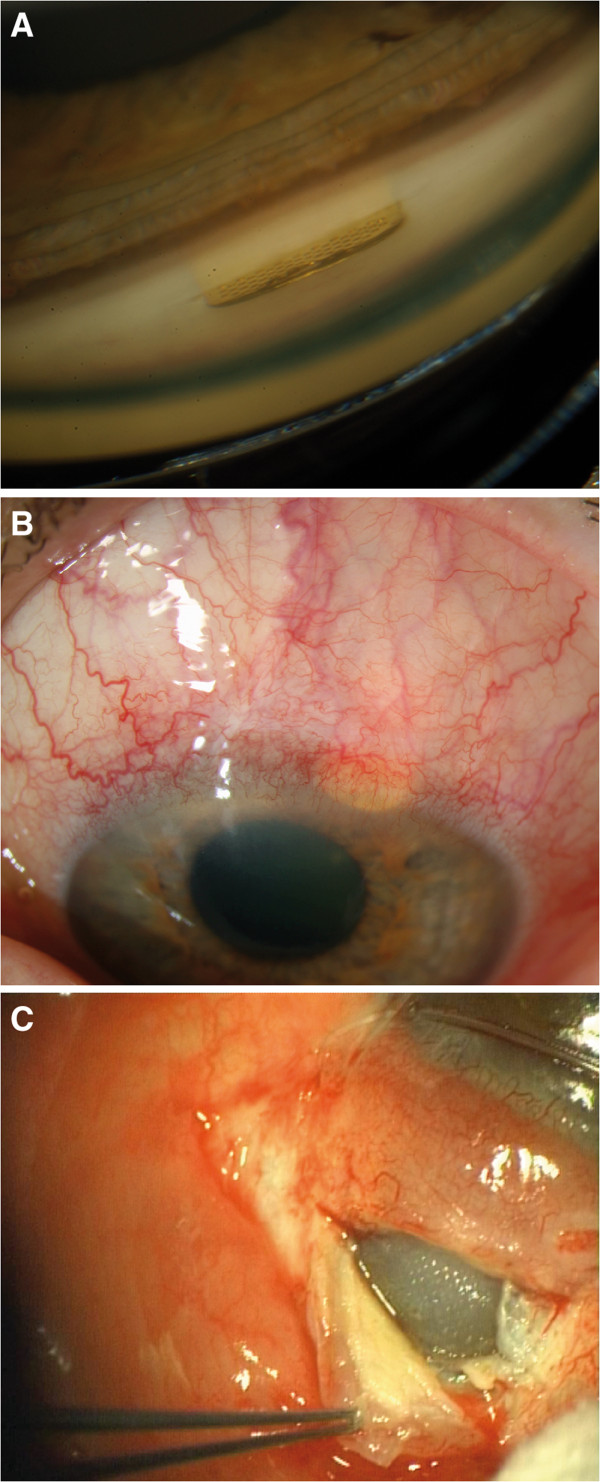
**Clinical photographs of the GMS+.(A, upper)** GMS+ gonioscopically one month after surgery. **(B, middle)** GMS+ one year after surgery with conjunctival hyperemia, deep and superficial corneal neovascularization and rubeosis iridis in a patient with POAG. **(C, lower)** After explanation of the GMS+, the color of the choroid changed from dark red-blue to gray.

Patients were operated on under general anesthesia or local anesthesia using a sub-Tenon injection. A fornix-based conjunctival flap was fashioned, followed by cautery of episcleral vessels. In 4 of 31 eyes mitomycin C (0.2 mg/ml) was applied to the sclera by means of a sponge for 2 minutes. Then the sclera was rinsed extensively with balanced salt solution. A 3 × 3 mm triangular, full-thickness, scleral flap was created 2 mm posterior to the limbus to expose the supraciliary space. The AC was entered at a plane of 90% scleral thickness using a 2.8 mm knife. Dissection into the suprachoroidal space posteriorly for 2 to 3 mm was done using hypromellose injection (AT.Viscose®) from Acri.Tec (Hennigsdorf, Germany). Next, the anterior segment of the GMS+ was introduced into the AC and the posterior segment was placed into the suprachoroidal space, ensuring that the implant was placed so that 1.0 to 1.5 mm of the GMS+ were visible in the AC. Two 10–0 nylon sutures were used to close the scleral wound securely. The conjunctiva was closed with two 8–0 vicryl sutures. All eyes were treated with Dexamethasone-Gentamycin eye drops 4 times daily for at least 2 weeks postoperatively. All patients underwent a GMS+ implantation by one surgeon (WK). After surgery the correct position of the GMS+ was confirmed by slit lamp examination in all cases (Figure 1A) and in 12 eyes (39%) the position was also confirmed by 50 Mhz ultrasound (Zeiss-Humphrey, System Model 840).

#### *Data evaluation*

The primary outcome measure was surgical success, defined as IOP less than 21 mmHg and greater than 5 mmHg and at least a 20% reduction of IOP from baseline IOP at least 6 months after GMS+ implantation. The postoperative use of IOP-lowering medications was evaluated, but was not considered in matters of surgical success. Criteria for failure were: 1) IOP outside the success range on one visit at least 6 months after GMS+ implantation, 2) serious complications at any time or 3) need for additional glaucoma surgery (except GMS+ repositioning) at any time. Serious complications included retinal detachment, endophthalmitis, suprachoroidal hemorrhage, low grade inflammation and newly developed rubeosis iridis. Intraoperative and postoperative complications were also recorded. For evaluation of the IOP lowering therapy, the number of different active ingredients were summed to a maximal of six. The 6 different active ingredients were beta blocker eye drops, sympathomimetic eye drops, parasympathomimetic eye drops, carbonic anhydrase inhibitor eye drops, prostaglandin analogue eye drops and carbonic anhydrase inhibitor tablets.

Patients were excluded from the study, if there was no failure but the follow-up time after surgery was less than 6 months. Analysis was performed with one eye per patient (n = 31 patients), including only the first eye that underwent GMS+ implantation. No patient received GMS+ implantation in both eyes simultaneously. The length of follow-up was defined as the time between the surgery and the next intervention (except GMS+ repositioning) or if there was no other surgery the last post-operative visit.

## Results

We identified 43 eyes of 38 patients, that received a GMS+ between February 2006 and June 2008. Five eyes were excluded, because of previous GMS+ implantation in the other eye. Another 7 eyes were excluded from the study, because the follow-up time after implantation, without additional glaucoma surgery, was less than 6 months. We included 31 eyes of 31 patients who were female, 15, and male, 16, with an age range from 28 to 82 years, mean age 60.0 ± 15.3 years. They were diagnosed with uncontrolled IOP and with the following types of glaucoma; primary open angle glaucoma (POAG), 17; pseudoexfoliation glaucoma (PEX), 5; secondary glaucoma (Sec), 4; pseudophakic closed-angle glaucoma (CAG), 3; and pigmentary glaucoma (Pigment), 2. In the group of secondary glaucoma, there were two patients with a known uveitis, one with a neovascular glaucoma and one with a history of silicone oil retinal surgery. Eighteen eyes were phakic and 13 eyes were pseudophakic. In 16 eyes (52%), the GMS+ was the first incisional glaucoma procedure. The remaining 15 eyes had unsuccessful prior procedures, as shown in Table 1. Before GMS+ implantation the cup-disc ratio, assessed by the surgeon, ranged from 0.4 to 1.0 (mean 0.88 ± 0.16) and the IOP ranged from 12 to 57 mmHg (mean 26.58 ± 10.14 mmHg). The sum of the number of different active ingredients in IOP lowering therapy ranged from 0 to 5 (mean 2.13 ± 1.61). The characteristics of the patients are summarized in Table 1.

**Table 1:** Summarized characteristics of the patients

**Age**^**a**^	**Glaucoma**	**Previous interventions**^**b**^	**Cup-disc ratio**	**Baseline IOP**^**c**^	**End IOP**^**d**^	**Follow up months**	**Next intervention**	**Outcome**^**e**^
70	Sec	P-T1	0.95	57 (4)	24 (0)	5.8	T	F(3)
70	POAG	none	0.80	34 (3)	44 (1)	8.9	T	F(1,3)
70	PEX	P	1.00	40 (3)	39 (3)	1.4	T	F(3)
60	POAG	none	0.95	16 (3)	21 (4)	2.2	T	F(3)
60	POAG	none	1.00	34 (0)	32 (3)	5.8	T	F(3)
80	POAG	P	0.85	18 (1)	17 (3)	5.0	T	F(3)
60	POAG	none	1.00	34 (0)	30 (3)	5.3	T	F(3)
70	POAG	P	0.80	17 (0)	17 (3)	7.9	T	F(1,3)
40	POAG	none	0.40	32 (4)	33 (1)	1.6	T	F(3)
80	CAG	P-C2	1.00	36 (2)	28 (3)	5.9	T	F(3)
30	POAG	T3-C1	0.95	17 (3)	18 (4)	2.8	T	F(3)
70	CAG	P-I-C3	1.00	28 (5)	17 (3)	3.4	T	F(3)
80	PEX	T3	1.00	26 (1)	44 (1)	9.3	T	F(1,3)
40	Pigment	none	0.90	30 (2)	29 (1)	4.5	T	F(3)
70	PEX	none	0.95	30 (4)	42 (1)	18.0	T	F(1,3)
80	CAG	P-I	0.80	25 (4)	28 (4)	1.0	T	F(3)
30	Sec	P	0.95	28 (2)	25 (0)	37.1	T-GMS ex	F(1,2,3)
50	POAG	none	1.00	16 (1)	17 (1)	12.2	T-GMS ex	F(1,3)
60	POAG	P-A-T2	1.00	20 (3)	34 (0)	1.2	T-GMS ex	F(3)
60	Sec	P-T1	1.00	24 (2)	50 (1)	14.2	B	F(1,3)
60	POAG	P-A3-T2	1.00	20 (1)	21 (2)	9.0	B	F(1,3)
80	PEX	P-T1	0.95	43 (1)	32 (0)	1.3	B	F(3)
60	Sec	C1	0.60	40 (5)	46 (1)	0.5	Cryo	F(3)
60	PEX	TA	0.40	27 (2)	28 (1)	5.3	P-GMS ex	F(2,3)
60	POAG	none	0.75	15 (0)	14 (0)	18.5	GMS ex	F(1,2,3)
60	POAG	none	0.80	14 (1)	12 (0)	12.2	GMS ex	F(1,2,3)
40	POAG	none	0.85	17 (0)	18 (2)	21.6	None	F(1)
50	POAG	T1	1.00	12 (2)	19 (0)	6.7	None	F(1)
50	POAG	T1	0.95	26 (0)	26 (0)	41.3	None	F(1)
40	Pigment	B-T1	0.80	20 (2)	24 (2)	6.6	None	F(1)
60	POAG	P	0.85	28 (5)	14 (0)	46.1	None	success

^a^Age in years when implanted (rounded half to even for de-identification).

^b^Previous interventions with number of interventions.

^c^Baseline IOP (mmHg) on the day before GMS+ implantation (sum of different active ingredients in IOP lowering therapy).

^d^IOP at the end of the follow-up (mmHg) (sum of different active ingredients in IOP lowering therapy).

^e^Outcome of GMS+ implant; F, failure (criteria met), for details see Methods.

*Abbreviations: Sec* secondary glaucoma, *P* pseudophakic, *T* trabeculectomy with mitomycin C, *I* iridectomy, *C* cyclodestructive intervention, *TA* trabecular aspiration, *A* argon laser trabeculoplasty, *B* Baerveldt implantation, *GMS ex* GMS+ explantation, *Cryo* Cryotherapy.

On the first day after GMS+ implantation the IOP ranged from 0 to 24 mmHg (mean 6.1 ± 5.1 mmHg). In three eyes the GMS+ was barely visible in the AC; so 3 to 10 days after initial implantation the position was revised to move the GMS+ 1.0 to 1.5 mm into the AC.

**Figure 2:**
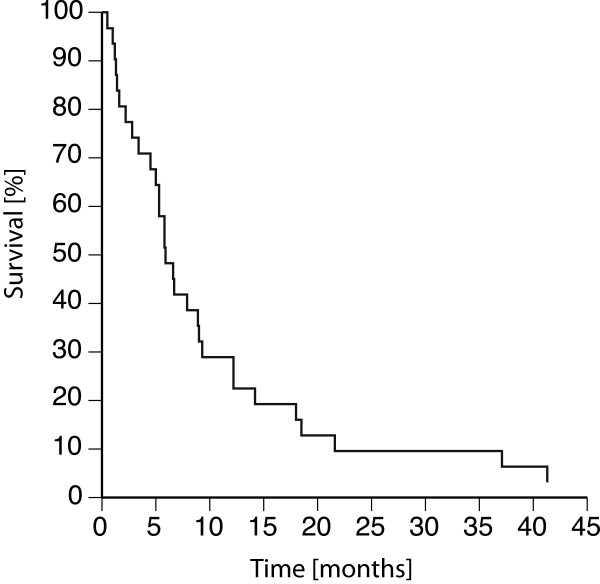
Kaplan-Meier survival curve for the outcome of 31 GMS+ implantations.

Altogether 30 of the GMS+ implanted 31 eyes met at least one of our criteria for failure; several met more than one criterion, as shown in Table 1. Within a mean of 7.3 ± 7.7 months another surgery was performed because of elevated IOP in 24 of 31 eyes (77%) (trabeculectomy with mitomycin C in 19 eyes, Baerveldt implantation in 3 eyes and one each of cryotherapy, and phacoemulsification with GMS+ explantation). Another surgery, GMS+ explantation, was required in 2 (6%) patients due to rubeosis iridis and low grade inflammation, without elevated IOP. The remaining 4 eyes, that met one of our criteria for failure, had an IOP reduction of less than 20% with comparable medication, as shown in Table 1. The survival curve is shown in Figure 2. The 1-year failure rate was 71% and the 2-year failure rate was 90%.

One eye had an IOP after the GMS+ implantation of 14 mmHg after 46.1 months of follow up and was considered a success. This patient had epithelial and stromal corneal edema and corneal neovascularization before GMS+ implantation.

For all 31 patients, the mean IOP at the end of the study was 27.19 ± 10.44 mmHg. The sum of the different active ingredients in IOP lowering therapy at the end of the study was 1.55 ± 1.39.

As shown in Table 1, six GMS+ were explanted. In two eyes with POAG the GMS+ was explanted because of elevated IOP without visible signs of chronic inflammation and combined with a trabeculectomy with mitomycin C. In one eye with POAG, newly diagnosed rubeosis iridis and a deep vascularisation surrounding the GMS+ (without elevated IOP) were the cause for the explantation of the GMS+ (Figure 1B). In one eye with secondary glaucoma due to uveitis, the GMS+ explantation was combined with a trabeculectomy with mitomycin C and indicated due to elevated IOP and newly diagnosed rubeosis iridis surrounding the GMS+. In one eye with POAG the GMS+ was explanted because of recurring pain and recurring mild intraocular inflammation without elevated IOP. In one eye with pseudoexfoliation glaucoma the GMS+ was explanted because of elevated IOP and newly diagnosed keratic precipitates. During implantation of the GMS+ the choroid had a dark red-blue color, but during explantation of the GMS+, the choroid was gray (Figure 1C).

## Discussion and conclusions

The surgical technique of the implantation of the GMS+ had no major operative complications. The GMS+ position was revised in 3 eyes, when the GMS+ was hardly visible without gonioscopy.

In our study the implantation of the GMS+ was not effective in reducing IOP. Most patients required additional glaucoma surgery within a few months. Melamed et al. [4] reported surgical success in 79% of their patients, although two thirds continued with antiglaucoma medications. Their mean IOP decreased from 27.6 ± 4.7 to 18.2 ± 4.6 mmHg, compared to our findings, from 26.6 ± 10.1 to 27.2 ± 10.4 mmHg after GMS+ implantation. Figus et al. [5] achieved a complete success in 5.5% and a qualified success in 67.3% of the eyes. We cannot explain the remarkable differences between the results of Melamed and Figus and our results, despite the following considerations. One reason could be the difference in surgical methods, as we used a full-thickness scleral flap. It seems to be important to close the scleral wound tightly, because the aqueous humor must be directed to the suprachoroidal space avoiding the formation of a bleb. The GMS+ we used had a larger lumen and should have worked provided better flow, in contrast to the device used by Melamed and Figus. Bending of the device could occur during insertion and would be another reason for failure. To account for this problem Figus performed 50-Mhz and Melamed performed 20-Mhz controls in all eyes, compared to our 50-Mhz ultrasound controls in just 39% of the eyes. Study design and length of follow up appear comparable in all three studies. Patient characteristics were comparable to Melamed, but Figus only included patients with failed glaucoma surgery. Another reason for the differences between our results and those of Melamed could be the definition of success and failure. As shown in Table 1, some of the patients had a baseline IOP lower than 22 mmHg, so we could not use an IOP of less than 22 mmHg as the only criteria for success. To be able to evaluate such patients, our IOP success range also required that the IOP decrease by 20%, which was not required by Melamed. In most of these cases, the surgeon decided to perform an additional glaucoma surgery, because the individual target IOP was not reached; the target IOP of most patients was about 12 mmHg. The individual decision of the surgeon was not controlled by our study design. Figus defined failure as an IOP >21 mmHg or less than 33% reduction of IOP on 2 consecutive follow-up visits after 3 months.

We classified 30 of 31 (97%) GMS+ implantations as failures. We excluded 7 of 38 patients because of a follow-up time of less than 6 months. Even if these patients were counted as successes, 30 of 38 (79%) would have been failures.

Agnifili et al. [6] examined explanted failed GMS+ and found connective tissue filling all the inner space and creating a thick fibrotic capsule surrounding the end of the device as the possible reason of failure. The GMS+ were explanted 6.8 ± 2.5 months after surgery, which is comparable to our findings with another surgery within 7.3 ± 7.7 months.

We [3] described the implantation of a silicone tube from the anterior chamber into the suprachoroidal space and found a reduced IOP in 70% of the cases after 8 months. In contrast, most of the eyes described here were unimproved after 8 months. In the reported study of GMS implants, all eyes had multiple prior glaucoma surgeries. In the study reported here, many patients had no prior surgeries and therefore should have been easier to treat. Suprachoroidal surgery seems to work better in glaucoma eyes with previously failed glaucoma surgery [3,5]. The cup-disc ratio in this study was 0.88 ± 0.16, therefore the target IOP of most patients was about 12 mmHg. Because the baseline IOP was 26.6 ± 10.1, a strong IOP-lowering surgical procedure was necessary in most cases.

Our surgical technique to implant the GMS+ was comparable to the implantation of the silicone tube into the AC and suprachoroidal space with the use of a scleral flap [3] and initially recommended by SOLX Inc., but differed from the surgical technique described by Melamed [4]. They simply incised the sclera and did not prepare a flap. A third surgical technique was described by the manufacturer, SOLX Inc. They recommend cutting the cornea peripherally near the limbus approximately 3.2 mm in length. They then initiate a Descemet’s plane dissection with a spatula and dissect along this plane posteriorly with a Bevel-Down crescent knife to create a pocket in the supraciliary space. They then insert the GMS+ in the pocket and within the supraciliary space. They then cut the Descemet obliquely in the direction of the limbus and move the GMS+ forward into the anterior chamber through the Descemet opening. Then the corneal wound is secured with one or two 10–0 nylon sutures. We preferred the surgical technique using a small flap to provide a better view of the choroid. We believe that with both other surgical techniques it is more likely to damage the choroid, as the visualization of the choroid is very limited.

The GMS’s design changed over time, for example channels were made larger. The first GMS had a height of about 44 μm (this one was used by Melamed [4] and Figus [5]), the next generation (GMS+) had a height of 68 μm (this one was used in our study), the latest generation (sGMS+) has a height of 80 μm. With increasing height, the size of the channels increased. As the likely mechanism that controls flow in the case of the GMS is the proximal pressure in the suprachoroidal space and not the negligible effects from the small luminal dimensions (diameter and length) we should have noticed equal IOPs and not lower IOPs because of an increased flow. Nevertheless also the lengths of the channels were reduced significantly from GMS+ to sGMS+. Another design change is the orientation of the GMS. The GMS+ should have the narrow part of the shunt in the anterior chamber and the sGMS+ should have the narrow part of the shunt in the suprachoroidal space.

The silicone tube we used earlier [3] had a round lumen with a diameter of 300 μm, which is much larger than the lumen of all of the GMS’s. Using this silicone tube from the anterior chamber to the suprachoroidal space we had a much greater success, compared to our results with the GMS+.

Theoretically we would prefer a shunt-design that is not dependant on the shunt lumen, but allows flow from the anterior chamber to the suprachoroidal space through the inner lumen, and also at the outside of the shunt, providing even more flow into the suprachoroidal space.

The CyPass implant (Transcend Medical, Menlo Park, California, USA) is a new microsurgically implantable device composed of biocompatible, non-degradable, polyimide material. The implant is inserted ab interno through a 1.5 mm clear corneal incision under intraoperative gonioscopy into the supracilliary space. It should create a controlled cyclodialysis and a permanent internal drainage into the suprachoroidal space. Initial clinical experience showed a low rate of surgical complications with concomitant decreases in IOP and/or glaucoma medications [7]. Probably the success rate of the CyPass device could be related to the cyclodialysis and using more than one device in the same eye could be useful. Obviously CyPass implant or other ab interno devices will have a lower rate of intraoperative complications and could substitute the GMS.

The change in color of the choroid, shown at explantation of the GMS+ (Figure 1C) could be a sign of filtration or a sign of chronic inflammation with reduced blood flow through the choroid.

Signs of chronic inflammation after trabeculectomy is a rare incident [8], and probably related to the prevalence of anterior uveitis in the population. We found in four of the GMS+ implanted eyes (13%) signs of chronic inflammation or new developed rubeosis iridis. The purity of the gold for the GMS+ is stated as 99.95% and a case report describing a foreign body made of gold with 8.25% copper did not irritate the eye for 9 years [9], so the reason for the chronic inflammation and new developed rubeosis iridis remains unknown and has to be further investigated.

## Competing interests

None of the authors has conflict of interest with the submission. WK received three complimentary GMS+’s and travel expenses to a lecture from SOLX Inc. The authors have full control of all primary data and agree to allow BMC Ophthalmology to review the data if requested.

## Authors’ contributions

AH wrote the draft paper. AH, SR and JFJ collected data and performed statistical analysis. WK performed the surgery. All authors read and approved the final manuscript.

## Pre-publication history

The pre-publication history for this paper can be accessed here:

http://www.biomedcentral.com/1471-2415/13/35/prepub
